# Plaque-type dura mater graft-associated Creutzfeldt-Jakob disease: an autopsied case report

**DOI:** 10.1080/19336896.2026.2635298

**Published:** 2026-02-28

**Authors:** Daisuke Tahara, Daichi Yokoi, Nao Tahara, Akio Akagi, Yuichi Riku, Jun Sone, Hiroaki Miyahara, Hirohisa Watanabe, Masahisa Katsuno, Yasushi Iwasaki

**Affiliations:** a Department of Neuropathology, Institute for Medical Science of Aging, Aichi Medical University, Nagakute, Japan; b Department of Neurology, Nagoya University Graduate School of Medicine, Nagoya, Japan; c Department of Neurology, Fujita Health University School of Medicine, Toyoake, Japan

**Keywords:** Case report, Creutzfeldt-Jakob, dura mater graft, MMiK, plaque

## Abstract

Clinicopathologically, dura mater graft-associated Creutzfeldt-Jakob disease (dCJD) presents as either a non-plaque or plaque type. Here, we report an autopsy case of plaque-type dCJD, supported by genetic and biochemical analyses. A 41-year-old man manifested right-hand paraesthesia. He had received a dural graft in the right parietal region following a traumatic acute subdural haematoma 27 years before symptom onset. The clinical course was slowly progressive. The patient became unable to walk independently 17 months after onset, showed cognitive decline at 22 months, and developed myoclonus and akinetic mutism at 24 months. Periodic sharp-wave complexes were never observed on electroencephalography throughout the disease course. He died 26 months after symptom onset. No mutations were identified in the prion protein (PrP) gene, and the codon 129 polymorphism was homozygous for methionine. Neuropathologically, mild to moderate spongiform changes with fine vacuoles, neuronal loss, and astrogliosis were observed in the brain and spinal cord. Degeneration was relatively severe in the limbic system, striatum, thalamus, and cerebellum, resembling the distribution pattern of VV2 sporadic CJD. Abnormal PrP deposition was broadly distributed consisting of synaptic, perineuronal, and plaque forms. In particular, intense PrP staining was observed throughout the spinal grey matter. Western blotting detected intermediate-type PrP in the brain and cervical cord, but not in systemic organs. Considering the clinical course and PrP staining in the spinal cord, PrP transmission is suggested to occur not directly from the transplanted dura mater to the central nervous system, but rather indirectly via a peripheral route.

## Introduction

Dura mater graft-associated Creutzfeldt-Jakob disease (dCJD) is an iatrogenic form of Creutzfeldt-Jakob disease (CJD) [1]. Cadaveric dural grafts were previously used for dural repair in neurosurgery [2]. Some recipients of grafts derived from humans with undiagnosed CJD later developed CJD after surgery (i.e., dCJD) [1]. There are two clinicopathological types of dCJD: non-plaque and plaque types [3]. Patients with the former type exhibit clinical courses identical to those of typical sporadic CJD and, neuropathologically, show synaptic-type prion protein (PrP) deposition without plaque formation [3,4]. In contrast, the latter type of dCJD follows an atypical clinical course, and its pathological hallmark is the presence of PrP plaques [3,4]. Approximately one-third of dCJD cases are of the plaque type [3]; however, detailed neuropathological descriptions of this form remain limited. Herein, we report an autopsied case of plaque-type dCJD characterized by distinctive lesion distribution together with biochemical and genetic analyses.

## Clinical summary

A Japanese male patient presented with paraesthesia in the right hand at 41 years of age. The patient had received a dura mater graft in the right parietal region 27 years earlier (at 14 years of age, in 1985) due to a traumatic acute subdural haematoma. The graft brand was Lyodura®. The patient had no relevant family history and had never travelled to or lived in the United Kingdom. The paraesthesia extended bilaterally to the forearms and lower legs after seven months. The patient had cervical spondylosis and underwent cervical laminoplasty 15 months after symptom onset. However, his symptoms progressively worsened, and he became unable to walk independently after 17 months. Furthermore, he gradually became unable to speak fluently. The patient was admitted to our hospital at 22 months after the onset.

At admission, in addition to paraesthesia, he exhibited disorientation, severe attentional deficits, and constructional disturbances. His Mini-Mental State Examination and Frontal Assessment Battery scores were 16 and 12, respectively. Other neurological findings included horizontal eye-movement disturbance, nystagmus, dysarthria, limb rigidity, bilateral Babinski reflexes, and limb and truncal ataxia. However, involuntary movements such as myoclonus were not observed. Cerebrospinal fluid analysis revealed elevated 14–3-3 and total tau protein levels (2348.8 µg/mL and >1300 pg/mL, respectively), and the RT-QuIC assay was positive for abnormal PrP. No mutations were identified in the PrP gene. Polymorphisms at codons 129 and 219 were homozygous for methionine and glutamate, respectively. Based on these findings, the patient was clinically diagnosed with dCJD.

At 23 months after onset, voluntary speech and movement further decreased. The patient became unable to take food orally, and nasogastric tube feeding was started. In addition, diffusion-weighted MRI revealed hyperintense signals in the bilateral caudate nuclei, putamen, globus pallidus, and thalamus at 23 months. The patient developed myoclonus and akinetic mutism at 24 months. Periodic sharp-wave complexes were not observed on electroencephalography throughout the disease course. He died of acute respiratory failure 26 months after onset, at 43 years of age.

## Methods

### Neuropathological examination

The left cerebrum, brainstem, cerebellum, and spinal cord were fixed in 20% formalin for four weeks. After cutting and trimming, the tissue blocks were soaked in 95% formic acid for one hour to inactivate prion infectivity. Sections from paraffin-embedded blocks were stained with haematoxylin and eosin, Klüver–Barrera, and Congo red. Immunohistochemistry for PrP was conducted using the mouse monoclonal antibody 3F4. We neuropathologically and semi-quantitatively evaluated degeneration and abnormal PrP deposition using previously described scales [5]: 0–4 for grey matter (0, no spongiform change or gliosis; 1, mild spongiform change or mild gliosis; 2, moderate spongiform change or moderate gliosis without apparent neuronal loss; 3, tissue rarefaction with hypertrophic astrocytosis or neuronal loss; and 4, status spongiosus), none–IV for white matter (none, no myelin pallor or gliosis; I, slight myelin pallor with mild gliosis; II, mild myelin pallor with hypertrophic astrocytosis; III, moderate myelin pallor with hypertrophic astrocytosis and foamy macrophages; and IV, severe myelin pallor and tissue rarefaction with axon loss), and (-)–(+++) for PrP deposition ((-), no staining; (±), little staining; (+), mild staining; (++), moderate staining; and (+++), strong staining) [5].

### Western blot analyses

Western blot analysis of protease-resistant PrP (PrP^Sc^) was performed on cryopreserved tissues. Analyses included samples from the right frontal, temporal, and occipital lobes; right thalamus and cerebellum; cervical spinal cord; and systemic organs such as the heart, liver, kidney, spleen, hilar lymph node, and iliopsoas muscle. The 3F4 antibody was used as the primary antibody. PrP^Sc^ of approximately 21 kDa and 19 kDa were designated as type 1 and 2, respectively [6,7].

### Transmission experiments

Brain homogenate derived from the present patient was inoculated into knock-in mice expressing human PrP with the polymorphism at codon 129 homozygous for methionine (Ki-129 M/M) or valine (Ki-129 V/V) [8].

### Ethical statement

This study was approved by the Research Ethics Committee of Aichi Medical University (approval number: 15–017), and was performed in accordance with the standards of the Declaration of Helsinki. Written informed consent was obtained from patients’ relatives before the autopsy.

Transmission experiments were approved by the Institutional Animal Care and Use Committee of Tohoku University (approval number: 2017 med-262), and performed in strict accordance with the Regulations for Animal Experiments and Related Activities at Tohoku University.

## Pathological findings

### Macroscopic findings

The brain weighed 1770 g before fixation. Surgical scarring consistent with a previous traumatic acute subdural haematoma was observed in the right parietal skull. The dura mater graft was firmly adherent to the skull. Mild cerebellar atrophy was observed. The cerebellar cortex was mildly atrophied, while the white matter and dentate nucleus were preserved. The cerebrum, brainstem, and spinal cord were also preserved.

### Microscopic findings

**Figure 1.**
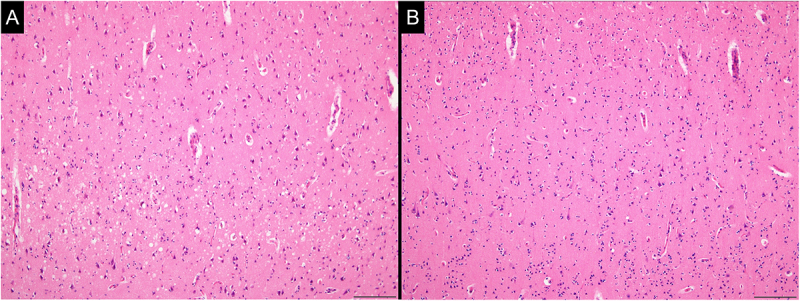
Representative images of spongiform changes.

**Figure 2.**
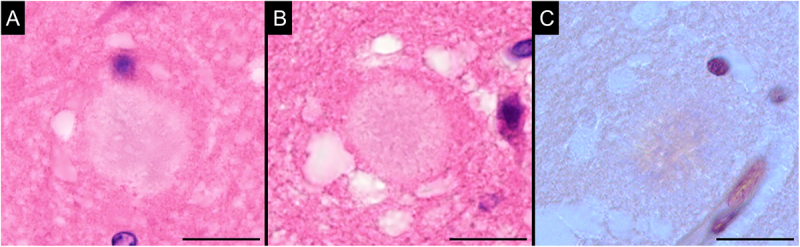
Representative images of kuru and florid plaques.

Mild-to-moderate spongiform changes with fine vacuoles in the neuropil were broadly observed in the cerebral cortex, basal ganglia, thalamus, cerebellar cortex, brainstem, and grey matter of the spinal cord (Figure 1). The vacuoles were round, well-defined, and varied in size. No lesions resembling status spongiosus were identified. In addition, numerous kuru and florid plaques were present in the neocortex on haematoxylin-eosin staining. The plaques exhibited a mild apple-green colour upon Congo red staining by observation using a polarizing microscope (Figure 2).

Mild to moderate spongiform changes with fine vacuoles were observed in the brain and spinal cord. The vacuoles had round shapes of varying sizes and did not tend to fuse. In the cerebral cortex, spongiform changes were most prominent in the deep layer, and these changes were more severe in the limbic system than in the neocortex. (Entorhinal cortex (A) and superior temporal cortex (B); haematoxylin-eosin staining; scale bars: 100 µm).

The kuru plaque has a round, mildly basophilic structure with a core (A), while the florid plaque has a similar structure surrounded by vacuoles (B). In this case, these plaques appeared a mild apple-green colour on Congo red staining, as observed with a polarizing microscope (C). (Superior temporal cortex (A), anterior cingulate cortex (B), and insular cortex (C); haematoxylin-eosin staining (A, B) and Congo-red staining with a polarizing microscope (C); scale bars: 10 µm).

Tables 1 and 2 show the degrees of degeneration. Degeneration in the neocortex ranged from 0 to 2, whereas that in the limbic system ranged from 1 to 3 (Table 1). Degeneration in the basal ganglia and thalamus was mild to moderate (Table 1). In the cerebellum, cortical degeneration ranged from 0 to 3, and degeneration in the white matter ranged from I to III. The dentate nucleus showed mild degeneration (Table 1). In the brainstem, degeneration of grey and white matter ranged from 0 to 2 and from I to III, respectively (Table 2). In the spinal cord, degeneration due to dCJD was observed only in the posterior horn (Table 2).

**Table 1.** Neuropathological evaluations of cerebrum and cerebellum.

Part	Degeneration	Abnormal prion protein deposition
Neocortex		
Middle frontal gyrus	1–2	(+)–(++)
Superior temporal gyrus	0–1	(+)
Superior parietal lobe	1–2	(++)
Striate cortex	0	(±)
White matter	none–III	(-)
Limbic system		
Hippocampus	2	(+++)
Parahippocampal gyrus	2	(++)
Cingulate gyrus	2–3	(+++)
Amygdala	1–2	(++)
White matter	none–I	(-)
Basal ganglia		
Caudate nucleus	2	(+++)
Putamen	2	(+++)
Globus pallidus	1	(++)
Subthalamic nucleus	1	(++)
Thalamus		
Medial nuclei	2	(+++)
Lateral nuclei	2	(+++)
Cerebellum		
Molecular layer	1–3	(+++)
Purkinje cell layer	0–3	(+++)
Granular layer	0–3	(+++)
White matter	I–III	(+)
Dentate nucleus	1	(+++)

0–4 indicates semi-quantitative evaluations of grey matter: 0, no spongiform change or gliosis; 1, mild spongiform change or mild gliosis; 2, moderate spongiform change or moderate gliosis without apparent neuron loss; 3, tissue rarefaction with hypertrophic astrocytosis or neuron loss; 4, status spongiosus. None–Ⅳ indicates semi-quantitative evaluations of white matter: none, no myelin pallor or gliosis; I, slight myelin pallor with mild gliosis; II, mild myelin pallor with hypertrophic astrocytosis; III, moderate myelin pallor with hypertrophic astrocytosis and foamy macrophages; IV, severe myelin pallor and tissue rarefaction with axon loss. The degree of abnormal prion protein deposition was semi-quantitatively assessed as follows: (-), no staining; (±), little staining; (+), mild staining; (++), moderate staining; (+++), strong staining.

**Table 2.** Neuropathological evaluations of brainstem and spinal cord.

Part	Degeneration	Abnormal prion protein deposition
Midbrain		
Substantia nigra	2	(+++)
Red nucleus	1	(++)
Superior colliculus	2	(+++)
Periaqueductal grey	2	(+++)
Cerebral peduncle	I	(±)
Pons		
Nucleus pontis	0	(++)
Locus ceruleus	0	(++)
Longitudinal pontine bundle	I	(-)
Superior cerebellar peduncle	I	(-)
Central tegmental tract	II	(+)
Medulla oblongata		
Inferior olivary nucleus	1	(+++)
Dorsal nucleus of vagus	0	(++)
Hypoglossal nucleus	0	(+)
Pyramid of medulla	III	(±)
Spinal cord		
Anterior horn	0	(+++)
Lateral horn	0	(+++)
Posterior horn	1	(+++)
Anterior funiculus	none	(±)
Lateral funiculus	none	(±)
Posterior funiculus	none	(+)
Anterior root	none	(-)
Posterior root	none	(+)

0–4 indicates semi-quantitative evaluations of grey matter: 0, no spongiform change or gliosis; 1, mild spongiform change or mild gliosis; 2, moderate spongiform change or moderate gliosis without apparent neuron loss; 3, tissue rarefaction with hypertrophic astrocytosis or neuron loss; 4, status spongiosus. None–Ⅳ indicates semi-quantitative evaluations of white matter: none, no myelin pallor or gliosis; I, slight myelin pallor with mild gliosis; II, mild myelin pallor with hypertrophic astrocytosis; III, moderate myelin pallor with hypertrophic astrocytosis and foamy macrophages; IV, severe myelin pallor and tissue rarefaction with axon loss. The degree of abnormal prion protein deposition was semi-quantitatively assessed as follows: (-), no staining; (±), little staining; (+), mild staining; (++), moderate staining; (+++), strong staining.

**Figure 3.**
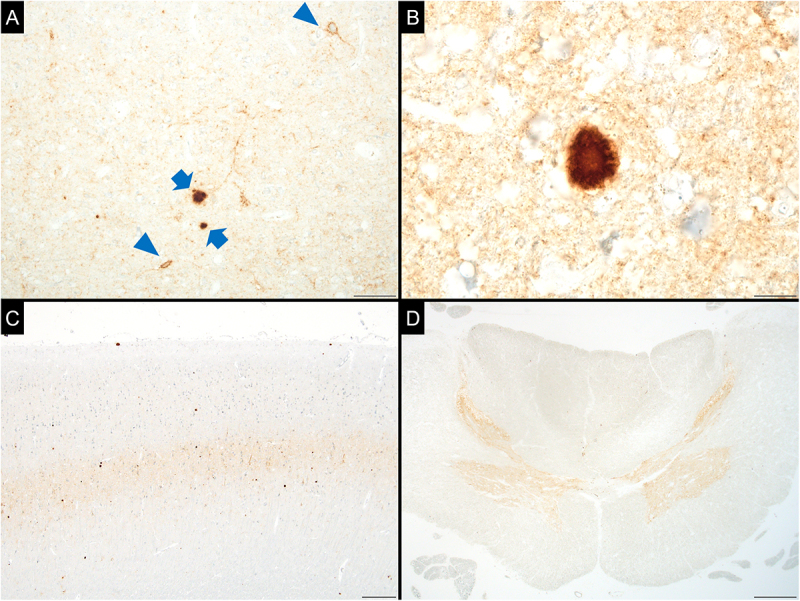
Abnormal prion protein deposition.

Abnormal PrP deposition was observed in both the synaptic type (i.e., diffuse microgranular deposits in the neuropil) and plaque form (Figure 3(A,B)). Perineuronal PrP deposition was also observed (Figure 3(A)). The extent of abnormal PrP deposition in the central nervous system is shown in Tables 1 and 2. Deposition occurred throughout the brain and spinal cord, with particularly intense PrP staining in the limbic system, striatum, cerebellum, and grey matter of the midbrain, medulla, and spinal cord (Tables 1 and 2). In the cerebral cortex, PrP deposition was most prominent in the deep layers (Figure 3(C)). In the spinal cord, strong PrP immunoreactivity was detected throughout the grey matter (Figure 3(D)). Furthermore, PrP deposition was also observed in the posterior funiculus and axons of the posterior root (Table 2). No abnormal PrP deposition was observed in the dura mater, dorsal root ganglia, olfactory bulb and tract, olfactory mucosa, or spleen. In the systemic organs, no PrP deposition was detected by immunohistochemistry.

Plaques (arrows) and perineuronal depositions (arrowheads) are observed along with diffuse microgranular depositions in the neuropil (synaptic-type deposition) (A). The plaque was examined at high magnification (B). In the cerebral cortex, prion protein deposition is most prominent in the deep layers (C). In the spinal cord, intense PrP deposition is observed throughout the grey matter, including the anterior, lateral, and posterior horns (D). (Superior temporal cortex (A), middle frontal gyrus (B, C), and upper cervical cord (D); immunohistochemistry for prion protein (3F4); scale bars: 50 µm (A), 10 µm (B), 200 µm (C), and 500 µm (D)).

Based on the clinical and histopathological findings described above, the patient was neuropathologically diagnosed with plaque-type dCJD.

### Results of western blot analysis

**Figure 4.**
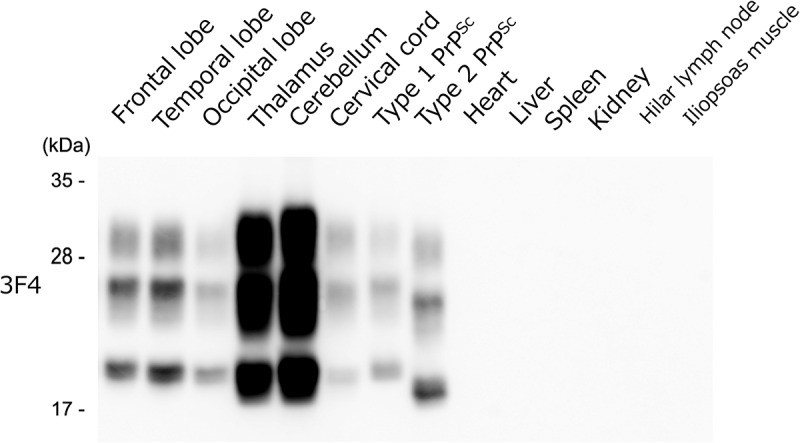
Results of Western blot analysis.

Western blot analysis of PrP^Sc^ identified an intermediate-type PrP^Sc^ with a molecular mass between 21 and 19 kDa in the brain and spinal cord. No PrP^Sc^ was detected in any of the systemic organs (Figure 4).

Protease-resistant prion proteins with molecular weights between 19 and 21 kDa (intermediate type) were detected in the cerebrum, cerebellum, and cervical spinal cord, but were not detected in the peripheral organs examined.

### Results of transmission experiments

Four out of five inoculated Ki-129 M/M mice developed prion disease with a mean incubation period of 638 ± 21 days. All five inoculated Ki-129 V/V mice developed prion disease with a mean incubation period of 316 ± 20 days.

## Discussion

The present case highlights two particularly important clinicopathological features of plaque-type dCJD. First, we demonstrated intense and widespread PrP deposition throughout the entire spinal grey matter, involving the anterior, lateral, and posterior horns. Such extensive involvement of the spinal grey matter has not been reported in sporadic CJD or non-plaque-type dCJD and is therefore considered a characteristic neuropathological finding of plaque-type dCJD. Second, disease onset with paraesthesia, together with PrP deposition in the posterior roots, posterior horns, and posterior funiculus of the spinal cord, suggests that prion infection may have occurred via a peripheral route, potentially through the dorsal roots, rather than by direct extension from the transplanted dura mater.

Nowadays, plaque-type dCJD is referred to as acquired CJD-MMiK (129 M/M genotype, type i PrP^Sc^, and kuru plaques), based on biochemical and genetic analyses [9]. Acquired CJD-MMiK can develop when the V2 CJD strain, which is associated with VV2 or MV2K sporadic CJD, is transmitted to individuals who are homozygous for methionine at codon 129 of the PrP gene [9]. In patients with CJD-MMiK, an intermediate-type PrP^Sc^ (i.e., with a molecular mass between 21 kDa [type 1 PrP^Sc^] and 19 kDa [type 2 PrP^Sc^]) is detected by Western blot analysis, and neuropathologically, kuru plaques are observed in the brain [9]. The clinical course of plaque-type dCJD is slowly progressive, with some neurological symptoms (e.g., myoclonus and akinetic mutism) appearing significantly later than those in the non-plaque-type form [4]. Additionally, periodic sharp-wave complexes on electroencephalography are significantly less frequent [4]. The clinical, genetic, and pathological findings of the patient presented in this report are consistent with the characteristics of plaque-type dCJD. The results of the transmission experiments were compatible with plaque-type dCJD, VV2 sporadic CJD, and MV2 sporadic CJD [10,11], suggesting that the present case was caused by transmission of the V2 CJD strain. It is likely that the transplanted dura mater was contaminated with the V2 CJD strain, which was subsequently transmitted to the patient.

One of the characteristic neuropathological findings in this case was the widespread, intense PrP deposition in the spinal grey matter. Deposition was observed in the anterior, lateral, and posterior horns, and PrP^Sc^ was detected in the cervical spinal cord by Western blot analysis. Few studies have reported spinal lesions in patients with plaque-type dCJD. One case report described moderate to severe degeneration of the grey and white matter; however, PrP deposition was not evaluated [12] (Table 3). Another study investigated PrP deposition in the spinal cord, peripheral nerves, and muscle [17]. In that study, three sporadic CJD and two dCJD cases all demonstrated synaptic PrP deposition in the posterior horn [17]. In the dCJD cases, one 42-year-old-male patient with a disease duration of 13 months exhibited plaque-type deposition in other regions of the central nervous system [17]. A comprehensive study of sporadic CJD cases also reported posterior horn-dominant PrP deposition [18]. Most cases showed little or no PrP staining in the anterior and lateral horns [18]. Only a few patients exhibited mild PrP deposition in these horns, accompanied by severe deposition in the posterior horn [18]. No cases have been reported to show moderate or more extensive PrP deposition in the anterior and lateral horns, such as that observed in the present case [18]. In sporadic and genetic CJD, as well as in non–plaque-type dCJD, there have been no reports of widespread PrP deposition throughout the spinal grey matter, as observed in the present case. This finding is therefore considered characteristic of plaque-type dCJD.

**Table 3.** Summary of studies describing ‘severe’ degeneration and/or spinal lesions in cases with plaque-type dura mater graft-associated Creutzfeldt-Jakob disease.

Authors	Age at onset, years	Sex	Disease duration, months	Areas presenting ‘severe’ or ‘marked’ degeneration	Spinal areas presenting degeneration	Abnormal PrP distribution
Lane et al. [12]	28	F	18	Thalamus, striatum, neocortex, cerebellum, brainstem, and spinal white matter.	Gray matter and lateral and anterior columns.	NA
Shimizu et al. (case 1) [13]	68	M	8	Thalamus, striatum, cerebellum^a^, and superior cerebellar peduncle.	NA	Gray matter^b^ and cerebellar white matter.
Shimizu et al. (case 2) [13]	68	F	17	Thalamus, striatum, and cerebellum.	NA	Gray matter^b^ and cerebellar white matter.
Kretzschmar et al. [14]	56 or 57	M	8	Thalamus, temporal lobe, and brainstem.	NA	Cerebrum and cerebellum.
Mochizuki et al. [15]	64	F	18	Cerebellar granular cell layer.	NA	Cerebral cortex^c^.
Wakisaka et al. [16]	25 or 26	F	10	Thalamus, cingulate gyrus, middle frontal gyrus, and dentate nucleus.	NA	Gray matter^d^ and cerebellar white matter.
Tahara et al. (present case)	41	M	26	None.	Posterior horn^e^.	Gray matter^f^ and cerebellar white matter^e^.

Abbreviation: NA, not available. ^a^Dentate nucleus and white matter. ^b^Gray matters in cerebrum, cerebellum, and brainstem. ^c^It is unknown whether other areas were investigated or not. ^d^Cerebral cortex, putamen, globus pallidus, thalamus, and cerebellar molecular layer. ^e^Details are indicated in Tables 1 and 2. ^f^Gray matters in the cerebrum, cerebellum, brainstem, and spinal cord.

Some mechanisms have been proposed for the propagation of PrP from the graft, including direct invasion from the graft to adjacent tissues and indirect spread via the cerebrospinal fluid, blood, or lymphatic system [19]. Based on the relationship between transplantation sites and initial symptoms, in non-plaque-type dCJD, PrP appears to propagate primarily through direct extension from the graft [19]. Importantly, in plaque-type dCJD, graft sites and initial symptoms are not statistically correlated, suggesting that different propagation pathways may be involved compared with non-plaque-type dCJD [19]. In this case, the initial symptom was paraesthesia in the right hand, although the dural graft had been placed in the right parietal cerebrum. Additionally, no abnormal signal intensity was observed on diffusion-weighted MRI of the brain, including the right parietal region, until 23 months after onset. Strong PrP deposition was observed throughout the spinal grey matter. No PrP deposition was detected in the systemic organs. Furthermore, Western blot analysis revealed the presence of PrP^Sc^ in the central nervous system, including the spinal cord, but not in systemic organs. The patient’s initial symptoms may have originated from involvement of the dorsal horn of the spinal cord. However, considering previous studies on spinal cord pathology [18], whereas PrP deposition is observed in the dorsal horn from the early stages of the disease, but even over a long disease course, it is unlikely to progress to the extensive and intense deposition throughout the spinal grey matter observed in this case. Furthermore, PrP deposition was observed in the posterior funiculus and posterior roots. Therefore, we speculate that PrP transmission may not have occurred through direct spread from the transplanted dura mater to the central nervous system, but rather occurred indirectly via a peripheral route.

Degeneration was observed throughout the brain and spinal cord (Tables 1 and 2). However, severe lesions described as scale 4 or IV in the grey or white matter, respectively (i.e., status spongiosus or severe myelin pallor and tissue rarefaction with axonal loss), were not observed (Tables 1 and 2). In non-plaque-type dCJD, evaluated neuropathologically using the same evaluation method, severe lesions were found in the neocortex, striatum, thalamus, and cerebellum [5]. ‘Severe’ or ‘marked’ degenerative lesions were previously reported in plaque-type dCJD [12–16] (Table 3); however, the severity in those cases appears milder than scale 4 changes, judging from the figures presented in those reports [13,15,16]. Otherwise, it was difficult to assess the degree of lesions in reports that did not include figures [12,14]. One neuropathological characteristic of patients with plaque-type dCJD would be that they generally exhibit milder degeneration than those with non-plaque-type dCJD.

Regarding lesion distribution, degeneration was relatively prominent in the limbic system and cerebellum (Tables 1 and 2). In addition, the striatum and thalamus showed clear evidence of degeneration (Table 1). A similar distribution pattern has been reported previously [13]. VV2 and MV2 subtypes of sporadic CJD display severe lesions in the limbic cortex and subcortical grey matter structures [7]. In particular, the lesion distribution in the present case resembled that of VV2 sporadic CJD (e.g., spongiosis was more pronounced in the hippocampal and parahippocampal regions than in the neocortex, and was especially marked in the cerebellum) [7]. Exceptions to this distribution pattern may also exist [15,16]; however, we believe that lesion distribution in plaque-type dCJD reflects the source of infection. In this case, the graft may have been contaminated with VV2 sporadic CJD.

The strengths of this case report include the reliability of diagnosis supported by neuropathological, biochemical, and genetic analyses. Furthermore, we presented detailed semi-quantitative pathological evaluations suggesting the characteristics of plaque-type dCJD and propagation pathway of PrP. A limitation of this case report is the rarity of dCJD. The reported longest incubation period is 30 years, and in many dCJD cases, transplantation of dura mater graft was performed between 1983 and 1987 [20]. We speculate that patients newly diagnosed with dCJD will be very rare in the future. Thus, it is difficult to conduct further comprehensive investigations in many plaque-type dCJD cases.

In summary, we reported an autopsy case of plaque-type dCJD consistent with acquired CJD-MMiK. PrP^Sc^ was detected in the brain and spinal cord, but not in systemic organs. Based on the clinical course and PrP deposition in the spinal cord and dorsal roots, we propose an indirect peripheral route of PrP transmission in plaque-type dCJD. Furthermore, degeneration was widely distributed throughout the brain and spinal cord, but was generally mild, with a pattern resembling that of VV2 sporadic CJD.

## Data Availability

The data are available from the corresponding author upon reasonable request.

## Abbreviations

CJD: Creutzfeldt-Jakob disease
dCJD: dura mater graft-associated Creutzfeldt-Jakob disease
MMiK: 129 M/M genotype, type i PrP^Sc^, and kuru plaques
PrP: prion protein
PrP^Sc^: protease-resistant PrP

## References

[1] Brown P, Brandel JP, Sato T, et al. Iatrogenic Creutzfeldt-Jakob disease, final assessment. Emerg Infect Dis. 2012;18(6):901–907. doi: 10.3201/eid1806.120116 22607808 PMC3358170

[2] Dong RP, Zhang Q, Yang LL, et al. Clinical management of dural defects: a review. World J Clin Cases. 2023;11(13):2903–2915. doi: 10.12998/wjcc.v11.i13.2903 37215425 PMC10198091

[3] Yamada M, Noguchi-Shinohara M, Hamaguchi T, et al. Dura mater graft-associated Creutzfeldt-Jakob disease in Japan: clinicopathological and molecular characterization of the two distinct subtypes. Neuropathology. 2009;29(5):609–618. doi: 10.1111/j.1440-1789.2008.00987.x 19659940

[4] Noguchi-Shinohara M, Hamaguchi T, Kitamoto T, et al. Clinical features and diagnosis of dura mater graft associated Creutzfeldt Jakob disease. Neurology. 2007;69(4):360–367. doi: 10.1212/01.wnl.0000266624.63387.4a 17646628

[5] Iwasaki Y, Mimuro M, Yoshida M, et al. Clinicopathologic characteristics of five autopsied cases of dura mater-associated Creutzfeldt-Jakob disease. Neuropathology. 2008;28(1):51–61. doi: 10.1111/j.1440-1789.2007.00847.x 18181835

[6] Parchi P, Castellani R, Capellari S, et al. Molecular basis of phenotypic variability in sporadic Creutzfeldt-Jakob disease. Ann Neurol. 1996;39(6):767–778. doi: 10.1002/ana.410390613 8651649

[7] Parchi P, Giese A, Capellari S, et al. Classification of sporadic Creutzfeldt-Jakob disease based on molecular and phenotypic analysis of 300 subjects. Ann Neurol. 1999;46(2):224–233. 10443888

[8] Kobayashi A, Asano M, Mohri S, et al. Cross-sequence transmission of sporadic Creutzfeldt-Jakob disease creates a new prion strain. J Biol Chem. 2007;282(41):30022–30028. doi: 10.1074/jbc.M704597200 17709374

[9] Kobayashi A, Parchi P, Yamada M, et al. Neuropathological and biochemical criteria to identify acquired Creutzfeldt-Jakob disease among presumed sporadic cases. Neuropathology. 2016;36(3):305–310. doi: 10.1111/neup.12270 26669818

[10] Kobayashi A, Parchi P, Yamada M, et al. Transmission properties of atypical Creutzfeldt-Jakob disease: a clue to disease etiology? J Virol. 2015;89(7):3939–3946. doi: 10.1128/jvi.03183-14 25609817 PMC4403436

[11] Kobayashi A, Iwasaki Y, Otsuka H, et al. Deciphering the pathogenesis of sporadic Creutzfeldt-Jakob disease with codon 129 M/V and type 2 abnormal prion protein. Acta Neuropathol Commun. 2013;1:74. doi: 10.1186/2051-5960-1-74 24252157 PMC3833290

[12] Lane KL, Brown P, Howell DN, et al. Creutzfeldt-Jakob disease in a pregnant woman with an implanted dura mater graft. Neurosurgery. 1994;34(4):737–739; discussion 739–740. doi: 10.1227/00006123-199404000-00026 8008176

[13] Shimizu S, Hoshi K, Muramoto T, et al. Creutzfeldt-Jakob disease with florid-type plaques after cadaveric dura mater grafting. Arch Neurol. 1999;56(3):357–362. doi: 10.1001/archneur.56.3.357 10190828

[14] Kretzschmar HA, Sethi S, Földvári Z, et al. Iatrogenic Creutzfeldt-Jakob disease with florid plaques. Brain Pathol. 2003;13(3):245–249. doi: 10.1111/j.1750-3639.2003.tb00025.x 12946015 PMC8095897

[15] Mochizuki Y, Mizutani T, Tajiri N, et al. Creutzfeldt-Jakob disease with florid plaques after cadaveric dura mater graft. Neuropathology. 2003;23(2):136–140. doi: 10.1046/j.1440-1789.2003.00489.x 12777102

[16] Wakisaka Y, Santa N, Doh-Ura K, et al. Increased asymmetric pulvinar magnetic resonance imaging signals in Creutzfeldt-Jakob disease with florid plaques following a cadaveric dura mater graft. Neuropathology. 2006;26(1):82–88. doi: 10.1111/j.1440-1789.2006.00638.x 16521484

[17] Ishida C, Okino S, Kitamoto T, et al. Involvement of the peripheral nervous system in human prion diseases including dural graft associated Creutzfeldt-Jakob disease. J Neurol Neurosurg Psychiatry. 2005;76(3):325–329. doi: 10.1136/jnnp.2003.035154 15716520 PMC1739566

[18] Iwasaki Y, Yoshida M, Hashizume Y, et al. Neuropathologic characteristics of spinal cord lesions in sporadic Creutzfeldt-Jakob disease. Acta Neuropathol. 2005;110(5):490–500. doi: 10.1007/s00401-005-1076-7 16175355

[19] Sakai K, Hamaguchi T, Noguchi-Shinohara M, et al. Graft-related disease progression in dura mater graft-associated Creutzfeldt-Jakob disease: a cross-sectional study. BMJ Open. 2013;3(8):e003400. doi: 10.1136/bmjopen-2013-003400 PMC3753481 23975105

[20] Ae R, Hamaguchi T, Nakamura Y, et al. Update: dura mater graft-associated Creutzfeldt-Jakob disease - Japan, 1975–2017. MMWR Morb Mortal Wkly Rep. 2018;67(9):274–278. doi: 10.15585/mmwr.mm6709a3 29518068 PMC5844283

